# X-Ray induced cataract is preceded by LEC loss, and coincident with accumulation of cortical DNA, and ROS; similarities with age-related cataracts

**Published:** 2010-08-06

**Authors:** William Pendergrass, Galynn Zitnik, Ryan Tsai, Norman Wolf

**Affiliations:** Department of Pathology, University of Washington, Seattle, WA

## Abstract

**Purpose:**

To compare age-related cataractous (ARC) changes in unirradiated mice lenses to those induced by head-only X-irradiation of 3 month-old mice.

**Methods:**

lens epithelial cells (LECs) as well as partially degraded cortical DNA were visualized in fixed sections using 4',6-diamidino-2-phenylindole (DAPI) staining, and in fresh lenses using the vital stain Hoechst 33342. reactive oxygen species (ROS) activity was also visualized directly in fresh lenses using the vital dye Dihydrorhodamine (DHR). In fixed lenses an antibody specific for 8-OH Guanosine (8-OH-G) lesions was used to visualize DNA oxidative adducts from ROS damage. Alpha smooth muscle actin was visualized using specific antibodies to determine if myofibroblasts were present. Fluorescence was quantified using Laser Scanning Confocal Microscopy (LSCM). The degree of lens opacity and cataract formation was determined by slit lamp, or from digitalized images of light reflections taken with a low magnification light microscope.

**Results:**

Using DNA- and ROS-specific vital fluorescent dyes, and laser scanning confocal microscopy we have previously described 4 changes in the aging rodent lenses: 1) a significantly decreased density of surface LECs in lenses from old compared to younger mice and rats; 2) a very large increase in retained cortical nuclei and DNA fragments in the secondary lens fibers of old rodent lenses; 3) increased cortical ROS in old rodent lenses; 4) increased cataract concomitantly with the cortical DNA and ROS increases. In the current study we report that these same 4 changes also occur in an accelerated fashion in mice given head-only X-irradiation at 3 months of age. In addition to vital staining of fresh lenses, we also examined sections from fixed eyes stained with DAPI or hematoxylin and eosin (H&E) and found the same loss of surface LECs and accumulation of undigested nuclei and debris in secondary lens fibers occur with age or following X-irradiation. In addition sections from fixed-eyes were examined for ROS damage to DNA with antibodies specific for 8-OH-G lesions. The frequency of 8-OH-G lesions increased dramatically in lenses from old unirradiated mice over 24 months of age, and similarly in X-irradiated lenses by 9–11 months post irradiation. The accumulation of cortical nuclei was not the result of conversion or invasion by myofibroblasts as tested by antibodies to a marker for such cells, alpha smooth muscle actin.

**Conclusions:**

X-irradiation damage induces a large decrease in surface LECs over a period of 3–11 months post X-irradiation of young mice. These changes are similar in extent to those seen in 24–29 months-old control mouse lenses with age-related cataracts. In 24+ month-old unirradiated mice the secondary lens fibers are not able to degrade nuclei or nuclear DNA efficiently and accumulate large numbers of cortical nuclei and nuclear fragments as well as ROS and 8-OHG lesions. X-irradiated lenses develop the same abnormalities in a more accelerated fashion. The extensive loss of LECS and accumulation of undegraded nuclei, ROS, and ROS damage may play a causal role in cataract generation in both unirradiated old mice and in previously irradiated young adult mice.

## Introduction

Age-related Cataract (ARC) is the main cause of blindness in the world today (see recent review [1]. Generally cataract is thought to result when the lens proteins or their environment become altered resulting in aggregation and precipitation of lens crystallins and other proteins forming reflective areas that block light transmission [2,3], and the generation of reactive oxygen species has been considered a possible causative agent [2,4-9]. Normally, the anterior central region of the lens is covered with nucleated amitotic lens epithelial cells (LECs). Lateral to this lies a ring of mitotic LECs, which subsequently migrate to the equator at the lens surface, elongate, and enter into the outer cortex where they continue a program of differentiation into secondary lens fibers (lens fibers accreted after adulthood). This differentiation process includes degradation and removal of lens organelles, and expression of lens crystallins and other lens specific proteins. As cell fiber cell accumulation progresses, serial layers of interiorized differentiated lens fibers are laid down burying the more secondary lens fiber cells deeper in the cortex. This process maintains the organization of the adult lens producing a clear “organelle free zone” (OFZ) in the inner cortex [10]. The maintenance of the OFZ is necessary for normal function and clarity of the adult lens [1,10-18]. Many things may interfere with the development of this highly organized structure of the lens that can lead to cataract formation [1-3,8]. It is not yet known exactly which alterations lead to ARC [1-3]. We have previously reported that an age-related decrease in the density of surface LECs occurs in old cataractous mouse and rat lenses, That is followed chronologically by a failure to degrade nuclei in the secondary lens fibers resulting in an abnormal accumulation of undegraded nuclei in the bow regions and spreading to the anterior and posterior cortices [19,20]. These cortical areas of abnormal LEC nuclei also express strong reactive oxygen species (ROS), and the appearance of the ROS and extended bow regions correlate to cataract incidence in the old animals [12,19-21].

If LEC loss and the accumulation of cortical nuclear fragments and ROS are instrumental in ARC progress, exogenous agents capable of damaging LECs, and inducing oxidative damage, would be expected to produce the same series of events in an accelerated fashion. Earlier workers have reported that radiation damaged LECs that survived the initial stress formed altered and damaged secondary lens fibers that were deflected to the posterior of the cortex [5,6,22-32]. To determine if ARC and radiation cataract progressed along similar lines, we administered 11 grays (one gray is the absorption of one joule of radiation, by one kilogram of matter) of head-only X-rays to three months old mice and followed the events for 11 months. We have published a preliminary report on the X-ray induced changes demonstrating a delayed but rapid onset of cataract 5–11 months following X-ray and summarizing some of the changes seen in the cataractous lenses [12]. In the present report we compare the LEC loss, abnormal expansion of the bow region, abnormal cortical ROS, and the formation of DNA oxidative adducts (8-Oxo-2'-deoxyguanosine abbreviated here as 8-OH-dG) in the lens cortex caused by head-only X-irradiation, to the same alterations seen in ARC. We, also, have examined these changes in fixed-whole eye sections in addition to vital staining of live metabolizing lenses to rule out artifactual changes that might occur during isolation of the lenses [12,19,20].

The dose of X- radiation, as used here, has been shown to induce primary damage in the LECs through induction of ROS, and/or by direct energy transfer to DNA and other macromolecules in the lens [12,33-38]. An important reason for our approach was to compare this form of oxidative damage to the lens and its expected induction of cortical cataracts to that of ARC, a condition that others and we have shown to be at least in part due oxidative damage events of biologic origin [21,39-43].

In the present study, X- irradiated lenses and control lenses are compared as a function of time for the 4 changes we previously observed in old lenses: 1) LEC loss, 2) decreased ability to degrade DNA in secondary lens fibers, 3) excessive ROS accumulation and ROS damage in the cortex, and 4) appearance of increasing opacities eventuating in cataracts.

## Methods

### Dyes and antibodies

All dyes were obtained from Molecular Probes, Inc. (Eugene, OR). Hoechst 33342 was kept as a 10 mM stock in water at 4 °C. Dihydrorhodamine 123 (DHR) was kept as a 5 mM stock in dimethyl sulfoxide (DMSO) at −20 °C. DAPI was in mounting medium from Vector (H1200; Vector laboratories, Burlingame, CA). Antibodies were obtained from the following sources: Alpha smooth muscle Actin was obtained from Abcam (#ab18460; Cambridge, MA); Goat polyclonal to 8-OH-dG from alpha from alpha diagnostic (#8OHG 12-S; San Antonio, TX); Rabbit polyclonal to αB-crystallin was also from Abcam (#ab13497); Lamin A/C was obtained from Cell Signaling technology (#2032; Danvers MA); antibody to aquaporin O was from Calbiochem (Long Beach, CA).

### Antibody staining of sections from fixed eyes for immunofluorescence analysis

In Dulbecco's Modified Eagle's Medium (DMEM) with 4-(2-hydroxyethyl)-1-piperazineethanesulfonic acid (HEPES) and 2% glucose, whole eyes were opened slightly at the site of entry of the optic nerve. Eyes were then transferred to 3.7% paraformaldehyde with 4% sucrose (pH 7.4) and fixed for 1 h. After washing in Trask’s PBS (2.7 mM KCl, 1.4 mM KH_2_PO_4_, 6.5 mM Na_2_HPO_4_, 0.14 M NaCl, pH 7.0) twice, the tissue was processed into paraffin blocks. Blocks were subsequently cut at a thickness of 10 microns. Slides with paraffin sections were deparaffinized in 3 changes of xylene, then washed for 5 min each in graded alcohols (100%, 95%, 80%, and 50%). The slides were then washed 2× with Trask’s phosphate buffered saline (TPBS) 5 min each, then rinsed with H_2_O and placed in Antigen Retrieval buffer (ARB, 10mM Sodium Citrate, pH6.0), heated to boiling in a microwaved and allowed to stand for 5 min. This heating step was repeated 2 more times and then the slides were allowed to cool in ARB for 40 min. Then the slides were rinsed with TPBS for 5 min and treated with 0.14% Triton X100, 10 mg/ml BSA (BSA; Sigma, St. Louis, MO) in TPBS for 5 min at room temperature. Then the slides were blocked for 60 min with 10 mg/ml BSA in PBS. The primary antibody was then added after a 1:200 dilution into blocking solution and left overnight at 4 °C. The slides were then washed with 3 changes of TPBS for 5 min each and then fluorescently labeled secondary antibody (1:200 dilution into blocking buffer) was added for 2 h at room temperature. The slides were washed 3 more times in TPBS, and the bound antibodies were then post-fixed with 3.7% paraformaldehyde for 5 min at room temperature, and then rinsed 2× more in TPBS for 5 min. Finally, salts were removed with a rinse of H_2_O), 75 μl of antifade mounting media (Vector laboratories, Burlingame, CA) was added and the slides coverslipped for analysis of fluorescence.

For the anti-8-OH-dG antibody, a DNA denaturation step was added after antigen retrieval. This consisted of 5 min treatment with 2N HCl at room temperature followed by 5 min in 1 M Tris base pH 8.0.

### Animals and tissues

Two sets of 12 irradiated and 12 non-irradiated control C57BL/6 female mice were used for the studies. The first set was irradiated at three months of age by head-only irradiation and were followed by slit lamp examinations (see below). At various ages, up to 11 months post-irradiation, members of this group were sacrificed by cervical dislocation, the lenses were immediately isolated by posterior excision from the extirpated eyeball and were stained with vital dyes and examined for oxidized DHR (rhodamine 123) which localizes in mitochondria, and Hoechst 33342 fluorescence. A second set of mice of equal number were irradiated at 3 month of age, and used to prepare “fixed-eye” paraffin sections for immunofluorescence studies at various ages. The mice in this group were sacrificed as above and the eyes fixed in 3.7% paraformadehyde for 60 min. at room temperature. The fixed eyes were then embedded in paraffin and 10 micron sections prepared for antibody staining. Females were used to avoid the stress and fighting damage often seen in separately weaned and caged males of this strain. These specific pathogen-free animals were purchased from Harlan Laboratories, Inc. (Indianapolis, IN) and housed four per cage and fed the standard maintenance Purina mouse diet 5015 (Purina Labs, St. Louis, MO). Humane protocols for maintenance and euthanasia from the University of Washington IACUC committee and the national AALAC organization were followed.

### Radiation source, dosage, and effects

Mice were irradiated as previously described [12]. A cabinet X-ray machine (Picker Company, Cleveland, OH; model 43855A, Series 209, cycles 50/60, Pre volts 115 with PKV 110 and Beryllium window) was used to irradiate mice placed 13 inches from the source. Delivery of 11 grays (Gy) of X-rays was at 5 mA, 100 kV over 5.5 min. This is a low energy delivery compared to the energy delivered by a 300 kV therapy machine or a Cesium source. Five mice at a time were placed on a circular rotating base with heads to the center and the remainder of the body including the tail covered with 3 mm of lead. Thus, only the heads were exposed to the x-radiation. Specialists from the University of Washington Environmental Health Division monitored the settings and delivery of radiation using a Victoreen meter. The mice were anesthetized with a mixture of ketamine (100 mg/ml) and xylazine (20 mg/ml) diluted with saline 12.6 fold to deliver anesthesia at 0.01 ml/gm of bodyweight. This was injected intraperitoneal (I.P.) just before the irradiation. The mice remained anesthetized for approximately 30 min. None of these animals in either the control or the head-irradiated groups showed any signs of distress, reduced physical activity, or reduced food intake during the study period from the beginning to the conclusion of the experiment 11 months following the irradiation. There was a general hair loss from the head that was obvious two weeks after irradiation with re-growth of melanin-free hair over the subsequent three to four weeks. Two mice developed sufficient subsequent scar tissue near one eye in each animal that, while not covering the eye, prevented an accurate slit lamp reading of cataract development on that side of the face.

### Lens preparation for vital staining and microscopy

For fixed sections whole eyes were opened slightly at the site of optic nerve entry to allow fixative to penetrate and placed in 3.7% paraformaldehyde for 60 min at room temperature. The fixed eyes were then processed into paraffin blocks and cut into 10 uM sections for staining and analysis.

For vital staining, whole eyes were placed corneal side down on sterile gauze and held in place by forceps. An incision was then made across the surface where the optic nerve enters the eye, and the sclera was pulled back to expose the lens. Debris from the ciliary body that remained attached to the equatorial plane of the lens was gently teased away with forceps before staining. As described separately below, 2 different microscopes were used, a low-power light microscope for whole lens pictures and cataract assessment and a Zeiss two-photon laser scanning confocal microscope (LSCM) was used to analyze lenses vitally stained with Hoechst 33342 and dihidrorhadamine (DHR) as previously described [19,44].

### Slit lamp examination and cataract scoring

As previously described [12] mice were hand-restrained without anesthesia, while the single examiner (N.W.) determined the opacity score using a SL-14 Kowa hand-held slit lamp (Kowa, Tokyo, Japan) in a dark room. Scoring was as in previous studies, on a basis of 0–4+ with half steps between the full numbers, and with one, two, and three representing progressive opacities and 4+ reserved for completely opaque mature cataracts. The examiner had no knowledge of the subgroups of animals (irradiated versus controls) presented to him and the selections made by the presenter were deliberately randomized. Dilation previous to slit lamp examination was with 1% tropicamide (Mydriacyl ophthalmic solution; Alcon, Fort Worth, TX).

### Lens opacity measurements with low-power reflecting light microscope

The intensity of light reflected from lens opacities was quantified by 2 methods; either by image analysis (Photoshop version 7.01; Adobe Systems, San Jose, CA) of digital photographs of the lenses taken with a low magnification reflected light microscope, or by using a separate reflected light channel on the LSCM (see next paragraph). The anterior side was determined from prior visualization of the Hoechst 33342 stained anterior epithelium under a fluorescent microscope. The lenses were then centered in the field of a low power light microscope (ZST; Unitron, Bohemia, NY), equipped with microscope adaptor (MM3XS; Martin Microscope, Easley, SC), and photographed with a digital camera using 16× magnification (Coolpix 5400; Nikon, Tokyo, Japan). Back lighting was provided by a tungsten light source (T-Q/FOI-1; Techni-Quip Hollywood, CA) with dual light guides positioned for side lighting. The lenses were then turned upside down, to photograph the posterior sides. The conditions were identical for young and old lenses photographed on each day.

### Staining fixed sections with antibodies for immunofluorescence

Paraffin sections were deparaffinized in xylene for 5 min repeated 2 more time, followed by 5 min in sequentially: 100%, 95%, 70%, and 50% ETOH, then 5 min in TPBS, pH 7.0. Then an antigen retrieval protocol was performed by putting the slides in 10 mM citrate pH 6.0, and heating to boiling in a microwave, leaving for 5 min, and repeating 2 more times. The slides were then allowed to cool in the citrate buffer, rinsed with water and PBS, then PBS with 0.14% triton X-100 for 5 min, then 5 more min in PBS only. For 8-OH-dG antibody staining, the DNA in cells on the slide was denatured by 5 min in 2 N HCl and rinsed for 5 min in 1 M tris base at pH 8.0 [45].Then the slides were blocked for 60 min in 1 mg/ml BSA in TPBS. The slides were then rinsed 2× with TPBS and 100 μl of primary antibody added diluted in blocking buffer 1:100 overnight at 4 °C. The next day the slides were rinsed 3× for 5 min each time with PBS, and incubated 90 min with secondary fluorescently tagged antibodies to the primary antibody. The slides were then rinsed 3× more for 5 min with PBS, and after a water rinse, mounted with mounting media containing DAPI (H1200; Vector laboratories). The slides were then analyzed on a Zeiss confocal microscope as described below.

### Staining of freshly isolated lenses with Hoechst 33342 and dihydrorhodamine (DHR)

The mouse lenses were viably stained with DHR and Hoechst 33342 as previously described [19]. Briefly, five μM DHR was added to the lenses already in 10 ml of lens medium (DMEM containing 25 mM HEPES with 7% fetal bovine serum) chilled to 0 °C for 45 min with mild agitation. Staining of the lenses with DHR was performed at 0 °C (but not frozen) to optimize differential staining of the abnormal ROS present in cataractous lenses with that produced by normal respiration of surface LECs at 37 °C. Cataract-related ROS was pre-existent in the lens cortex and did not require active metabolism. DHR staining of the mouse lens inclusions at 0 °C was just as intense as when done at 37 °C. Following DHR treatment, the lenses were rinsed briefly in lens medium, to remove free DHR, and stained with 10 uM Hoechst 33342 in lens medium at 37 °C for 15 min [19]. Freshly isolated metabolizing lenses were placed in chambered slides (Nalge Nunc International, Naperville, IL) in 2.0 ml of lens medium. The lenses were then photographed with a low power light microscope and chilled to 0 °C to stabilize the DHR and the DNA fluorescence during analysis with the LSCM. The doubly stained lenses were then kept on ice until analyzed with the LSCM. The fluorescence of the stained lenses was stable for at least 3 h on ice.

### LSCM analysis of vitally stained mouse lenses with Hoechst 33342, DHR, and DAPI, and immunofluorescence of antibody preparations

Two different confocal microscopes used to analyze stained lenses, a Zeiss 2-photon LSCM for vital stains and a Zeiss meta for analyzing fluorescent antibodies. The Zeiss 2-photon LSCM (model 510 NLO; Carl Zeiss MicroImaging, Inc., Göttingen, Germany) used for vitally stained whole lenses had the two-photon laser (Mira 900 IR femtosecond pulse laser 50 mW Titanium Sapphire; Coherent Inc., Santa Clara, CA) tuned to 750 nm for Hoechst 33342. Emitted light from the sample was passed through a primary dichroic mirror passing light only below 650 nm, a secondary optic passing light only below 490 nm, and finally a band pass filter near the Hoechst 33342 optima between 435 and 485 nm. The DHR (oxidized by ROS to rhodamine 123 inside the lens fibers) was analyzed using a 488-nm line from an argon laser (5 mW, run at 60% of full power; Lasertechnik GmbH, Berlin, Germany). Emitted light was selected with a primary dichroic that blocked the 488-nm exciting light, a secondary optic that transmits light waves shorter than 635 nm, a tertiary optic that transmits light waves longer than 490 nm, and a band-pass filter near the rhodamine 123 optima between the 500- and 550-nm wavelengths. In some cases, a separate reflection channel was set up that measured 543-nm light reflected from opacities. This was performed with the 543-nm excitation line from the Helium-Neon laser at 10% power, and detecting reflected light passing through a 500–550 -nm band-pass filter. Only one fluorochrome was excited at a time per frame scan to assure that only the desired probe was visualized. Unless otherwise specified, analyses were performed at both the anterior and posterior poles of the lens using a 10× objective and scanning 26 frames at 4 μm intervals from an area of the lens 1.3 mm in diameter beginning at the surface and ending 100 μm deep beneath the surface. Freshly isolated viable lenses were used for all vital staining. The background (fluorescence from the media surrounding the lens) was subtracted from the fluorescent intensities measured for the lens.

For the second LSCM used for analysis of antibody fluorescence in sections from fixed lenses, a Zeiss meta confocal microscope (Carl Zeiss MicroImaging, Inc.) was used. See description of microscope at the KECK facility at the University of Washington Medical School. DAPI was excited with a DPSS laser 405 nm line, Emitted light was passed thru a dichroic passing light <515 nm, then this light was filtered through a bandpass filter near the Hoechst 33342 optima between 420 and 480 nm. Green fluorescent-tagged antibodies (generally Alexa Fuor 488; Invitrogen, Eugene, OR) were analyzed using the 488-nm line of an argon laser (Lasertechnik GmbH). Emitted light was selected and transmitted through a 505–550 band-pass filter near the alexa fluor 488 optima. Antibody fluorescence from red Alexa Fluor 546, (Invitrogen) was illuminated with the Hellium-neon laser 543 excitation band with emitted light, then a dichroic passing light >490 nm, and finally thru a long pass 560 nm filter. In some cases, a separate reflection channel was set up that measured 543-nm light directly reflected from opacities. This was performed with the 543-nm excitation line from the Helium-Neon laser at 10% power, and detecting reflected light passing through a 500–550-nm band-pass filter. Only one fluorochrome was excited at a time per frame scan to assure that only the desired probe was visualized. For vital staining, analyses were performed at both the anterior and posterior poles of the lens using a 10× objective and scanning at 4 μm intervals from the anterior or posterior surface of the lens scanning 100 μm inwards. Freshly isolated viable lenses were used for all vital staining.

The LSCM images were downloaded to a computer (Macintosh; Apple Computer, Cupertino, CA) and analyzed with the NIH program Image J (developed by Wayne Rasband, National Institutes of Health, Bethesda, MD) as previously described [19].

### Widefield microscopes used in imaging

In some cases the fluorescence of fixed lens sections was analyzed using a Nikon Upright (Nikon Eclipse E600) with standard filtersets for DAPI (405 excitation), or alexafluor 488, or 546 antibodies, or bright-field photos using H&E staining (see description at the KECK website).

### Statistics

Regression coefficients between cataract scores and DNA and DHR were made using the linear regression program on computer (SPSS version 11 for Macintosh; SPSS Inc., Chicago, IL). Unless otherwise mentioned, a 1-tailed Mann–Whitney nonparametric (also from SPSS**)** was used for comparing the groups of young and old controls or X-irradiated mouse lenses.

### Normalization of DNA, DHR, and reflectance readings to young control subjects

Vital staining of mice lenses were made on two to four old lenses and two to four young lenses at a time (per LSCM session). To control for possible changes in staining or instrument sensitivities for the 3 months over which the vital stain measurements were made, the values of DNA, DHR, and reflectance for each lens was normalized to (divided by) that of 3–5 month young lens controls measured on the same day as the irradiated or older control mice. All results of vital staining were presented as a percentage of the young control animals unless otherwise stated. The data from the fluorescence of fixed sections are presented as absolute pixel intensities of the fluorescence analyzed using Image J 4.2.

## Results

**Figure 1:**
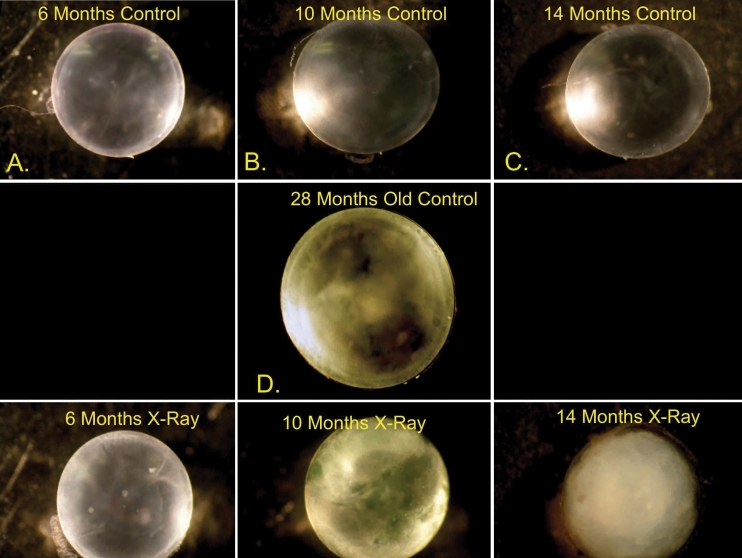
Typical appearance of freshly excised lenses from unirradiated control and X- irradiated mice at various ages. Unirradiated control lenses are depicted in panels **A**-**D**, and lenses from mice X-irradiated at 3 months of age are shown in **E**-**F**. The photos were taken through a dissecting scope using reflected light. The lenses were selected as representative of each age and treatment group.

**Figure 2:**
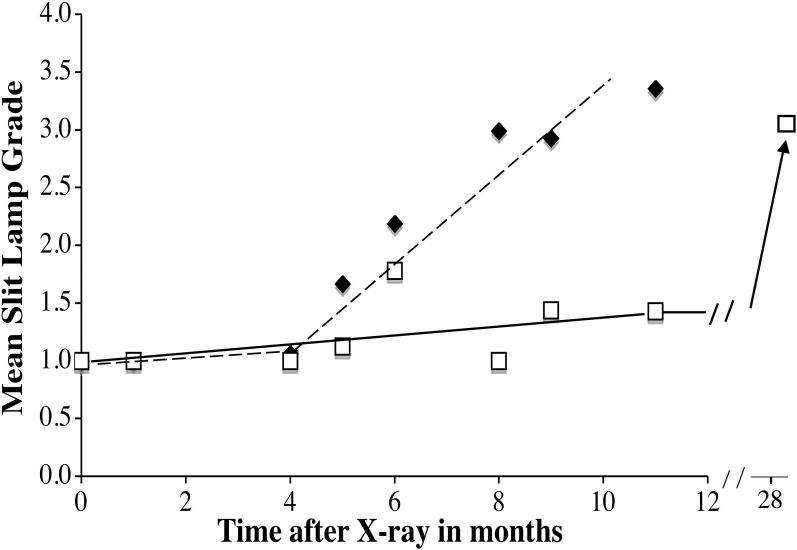
Average slit lamp grades of X-irradiated and control lenses. Slit lamp analysis was performed as described in Methods on the lenses from 12 mice at each time point. The slit lamp grades for lenses from X-irradiated mice are depicted as filled diamonds and control lenses as open squares. The value for the 28-month unirradiated controls is from [21]. The mice were X-irradiated at 3 months of age. The lines were fit by eye.

Figure 1 illustrates typical reflective properties of lenses at different times after X-irradiation and in age-matched unirradiated controls. Generally, no changes were observed in reflectance until at least 6 months post X-irradiation, when cataract development began accelerating. Cataract progression was greatly accelerated by X-irradiation although almost no cataracts were seen until 6 months post-irradiation, but by 11 months after X-irradiation, the cataract grades were similar to old control animals over 25 months of age (Figure 2).

**Figure 3:**
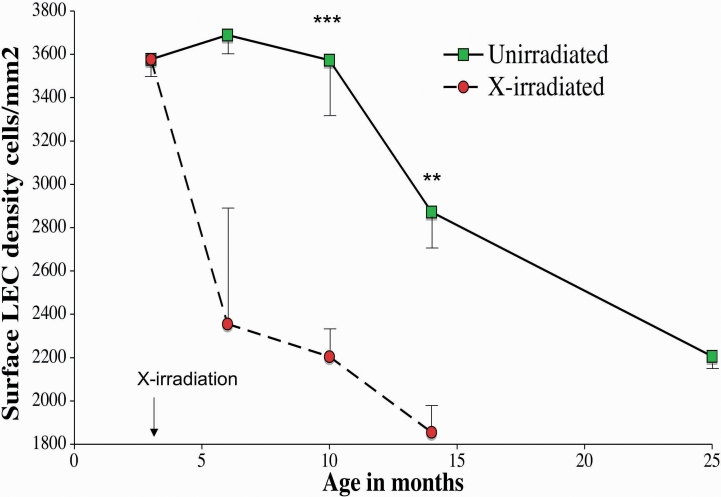
Decreases in mouse surface LEC cell density with age and X-irradiation using vital dye staining of whole lenses. The cell density (cells/mm^2^) was determined by counting nuclei stained with the vital dye Hoechst 33342 on the lens surface in unirradiated controls (green squares) and X-irradiated mouse lenses (red circles). X-Irradiation of these mice was at 3 months of age. The error bars represent the standard error of the means, and stars indicate a significant difference from age matched control values using 1 tail Mann–Whitney test (the p values: **, p<0.02, ***, p<0.002). Each point represents the mean of 4–6 mice.

**Figure 4:**
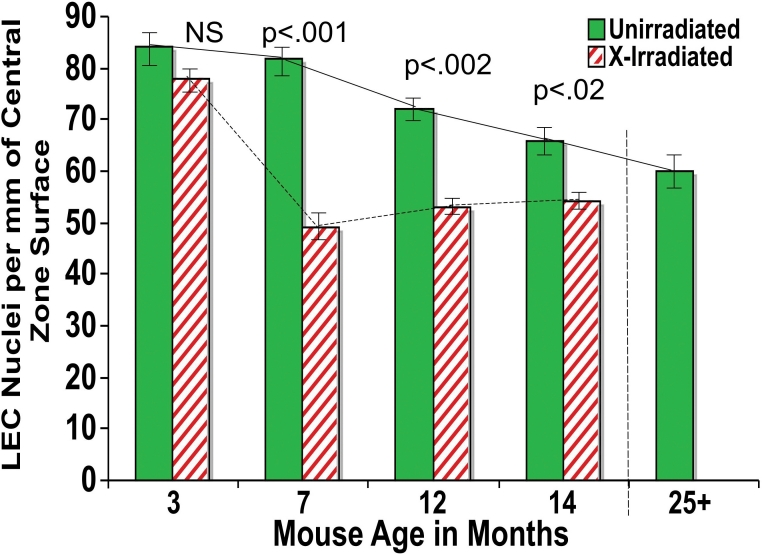
Changes in the density of surface LECs in fixed-eye sections. The LEC densities in the central zone of unirradiated control and X- irradiated lens sections were determined by counting the DAPI stained nuclei per mm of lens surface (see Methods). X-irradiation was at 3 months. The surface LEC nuclei/mm densities decreased with age (p<0.001 between unirradiated 3-month and 25 month-old mice lenses). The p values shown are for a comparison of the LEC densities in X-irradiated and age-matched controls at 7 months, 12 months, and 14 months using a single tailed Mann–Whitney test. The error bars are for the standard error of the mean. Each bar represents the mean for 6–8 mice.

The LEC density of the Central Zone was significantly reduced relative to unirradiated controls as measured by vital staining with Hoechst 33342. Figure 3 compares the number of LECs per mm^2^ of the vitally stained lens epithelia. A rapid decrease in the surface LEC density of the Central Zone occurred by 3 months following X-irradiation. Before X-irradiation was administered at 3 months of age, the LEC density was 3,641 nuclei/mm^2^, and by 3 months post X-irradiation (6 months of age), the cell density had dropped to 2,356 (63% of starting value), and by 11 months post irradiation, the cell density had dropped to 1,856 /mm^2^, (51% of young controls). This decrease in LEC density in X-irradiated mouse lenses was much more rapid  than the gradual decrease in the unirradiated controls to near the same level (2,090 cells/mm^2^ at 29 months of age). In both X-irradiated and controls this decrease in LEC density preceded cataract formation or other changes reported below. In a separate study of the LEC density, fixed-eye lens sections were also analyzed (Figure 4). The number of nuclei per linear mm of the Central Zone surface were counted using DAPI to stain the sections. This second study on fixed-eye sections produced similar results to those seen in the vital staining of whole lenses, indicating that the LEC densities of X-irradiated mice lenses were reduced significantly by 4 months after X-irradiation (p<0.001). In both studies the decrease in LEC density preceded cataract formation by several months both in X-irradiated and in old unirradiated control mice.

**Figure 5:**
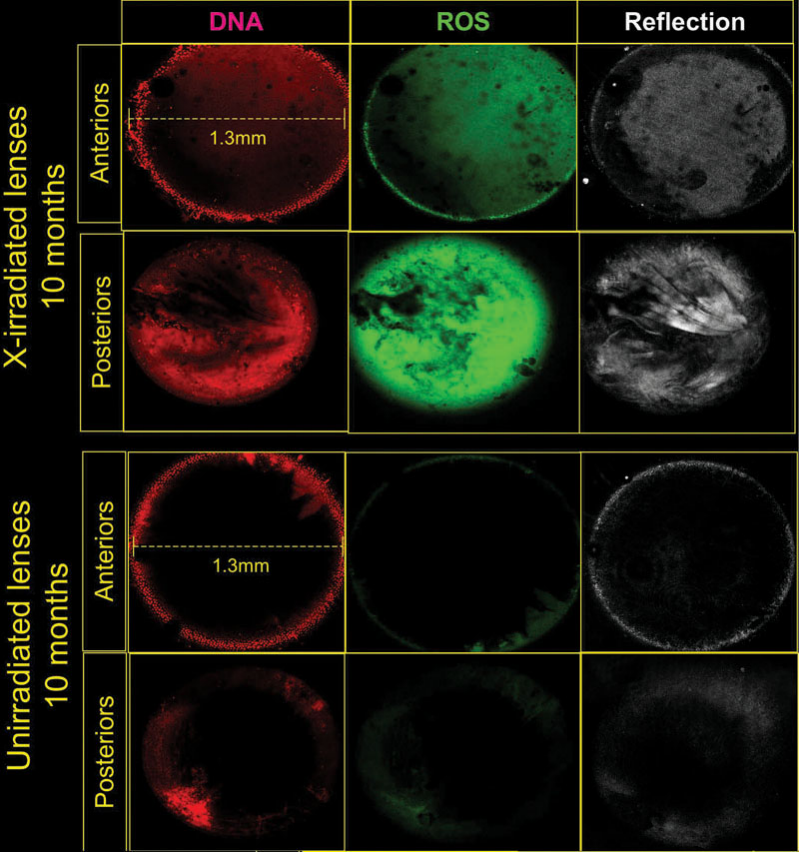
Typical vital staining of the anterior and posterior cortices of 10-month old mice lenses for DNA, ROS, and cataract. DNA (Hoechst 33342) in the lenses is shown in red, ROS (oxidized DHR) is shown in green, and cataract reflected light in white. The LSCM (10× objective) was focused 120 microns below the anterior poles of unirradiated control lenses (upper panel) and X-irradiated lenses (lower panel) at the same age. The donors of the lenses were 3 months old when irradiated and 10 months old when analyzed. The first column depicts the DNA fluorescence in red (Hoechst 33342 fluorescence), middle Column shows ROS in green (oxidized DHR). The right column shows light reflected from cataracts. Original magnification was 200×.

**Figure 6:**
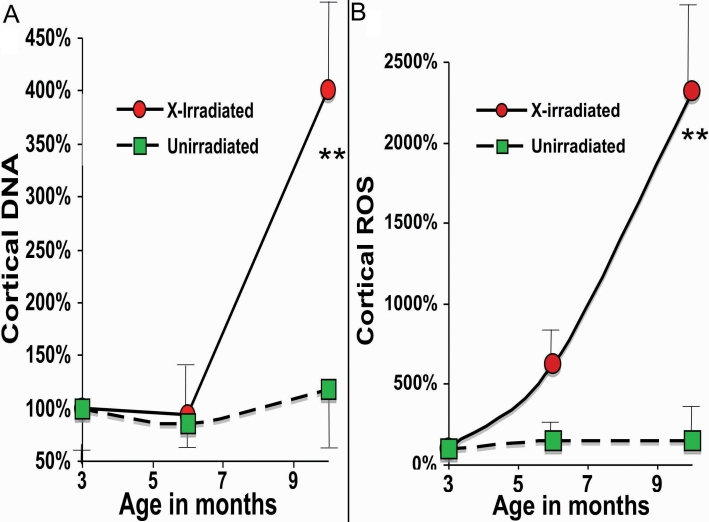
Increases in cortical DNA and ROS following X irradiation at 3 months of age. Vital staining of fresh lenses for ROS and DNA were carried out with the same stains shown described in the legend to Figure 5. The data are presented as per cent of 3-month controls (see Methods). Red circles are for lenses X-irradiated at 3 months and green squares are for unirradiated age-matched controls. Error bars represent standard error of the means, and stars indicate a significant difference from control values using 1 tail Mann–Whitney test (**, p<0.01). Each point represents the mean of 4–6 mice analyzed.

We previously reported that vitally stained lenses from old cataractous mice and rats contained large numbers of undegraded or partially degraded nuclei, and high levels of ROS in the lens cortex [19,20]. In Figure 5 typical examples of vital staining for cortical DNA (Hoechst 33342) and ROS (as oxidized DHR) in X-irradiated mouse fresh lenses and in age-matched unirradiated controls are compared. Figure 6 depicts the relative quantitative intensities of cortical DNA (DNA fragments and cytoplasmic DNA) using the vital dye method in X-irradiated mice and in age-matched unirradiated controls. Both the number of abnormal cortical nuclear fragments, as well as, the intensity of cortical ROS increased significantly in the X-irradiated lenses by 6 months post irradiation using the vital dye method.

**Figure 7:**
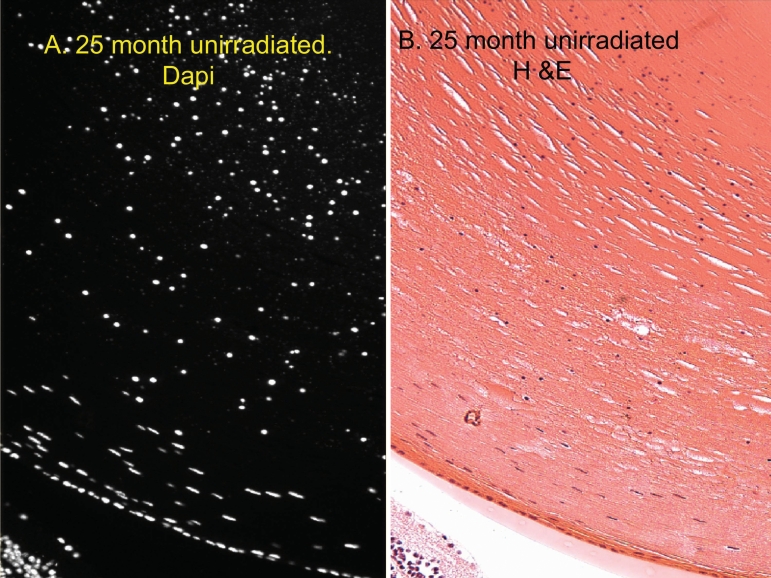
A comparison of nuclei and nuclear cortical fragments in a typical old-mouse eye section using both DAPI and H&E stains. DAPI (**A**) and H&E (**B**) staining are compared. All of the included nuclei stained with DAPI (white) were also stained blue by Hematoxylin. All other H&E and DAPI images demonstrated the same correspondence (not shown), but DAPI was much easier to score at lower magnification so was generally used in counting nuclei and nuclear fragments. Original magnification was 200×.

**Figure 8:**
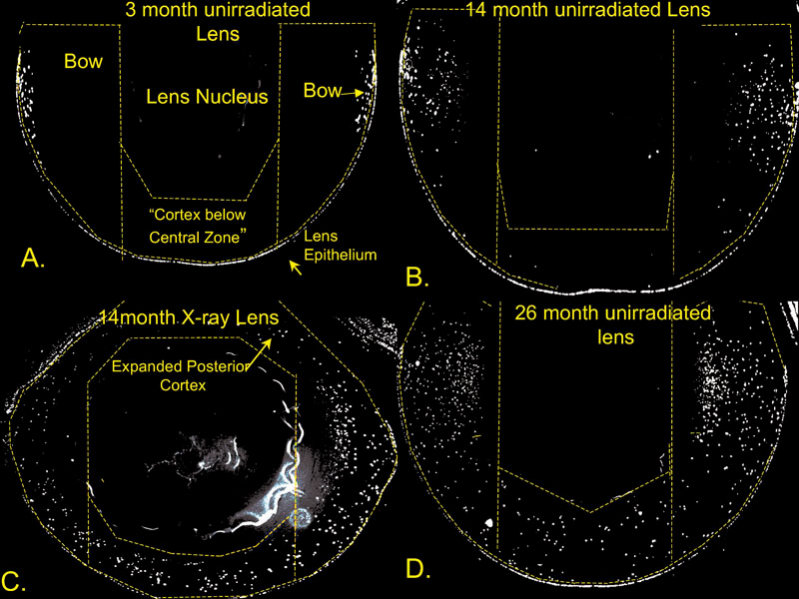
Typical images of DAPI-stained paraformaldehyde-fixed sections from lenses unirradiated control lenses and lenses from mice X-irradiated at 3-month of age. Panel **A **was from unirradiated 3-month controls, panel **B** was from 14 month unirradiated controls, panel **C** was from 14-month X-irradiated lens, and panel **D** was from a 26-month old unirradiated control. Each image was derived from a panorama taken with a 10× objective (see Methods). The dashed lines show the regions of the lenses used for counting DNA fragments in different regions of cortex (see Figure 9A,B). These include both bow regions, and the cortex below the central zone. The posterior cortex was included if nuclear fragments were present. The non-lens portions of the eye have been deleted for clarity. Original magnification was 200×.

**Figure 9:**
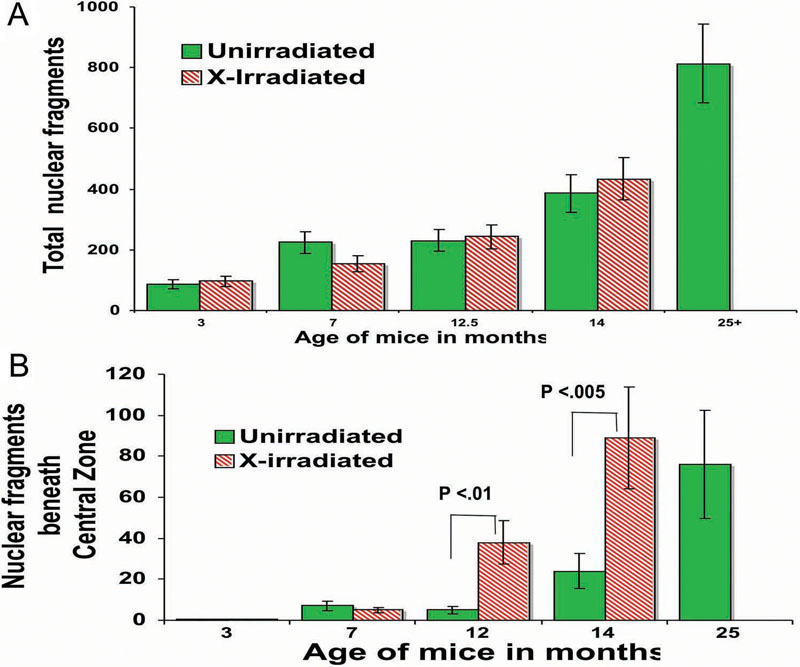
The number of total cortical nuclei in lenses from unirradiated control mice and mice irradiated at 3 months of age. Panel **A** depicts the number of total retained nuclear fragments in the whole cortex, and panel **B** shows the number of nuclei in the cortex beneath the Central Zone only. Control and X-irradiated mice lenses were fixed, sectioned, and stained with DAPI for DNA as described in the Methods. The total number of nuclear fragments was counted in 5–7 lenses from each age and/or radiation group except the 3 month-old X-irradiated group, which consisted of data from two animals. The number of total number of cortical nuclear fragments was not significantly greater in X-irradiated lenses than controls of the same age. However, the number of nuclear fragments found in inappropriate positions below the central zone of X-irradiated lenses (panel B) was significantly higher than age matched controls at 12 months X-rayed (p<0.01), and 14 months X-rayed (p<0.005). Original magnification was 100×. The p values were determined by a 1-tailed Mann–Whitney test.

We also compared the number of cortical nuclei present in fixed-eye lens sections using both DAPI and H&E stains. The H&E staining and DAPI staining were very similar with every DAPI-stained-nucleus also being stained by H&E (Figure 7). The DAPI was used for further analysis because it was brighter and permitted more rapid examination of the lens sections. Examples of fixed-eye sections from 3 month, 14 month, and 26-month-old unirradiated control lenses, and a 14-month-old X-irradiated lens (Figure 8C) are compared in Figure 8A-D. These sections were cut through the middle of the whole eye at right angles to the anterior surface to reveal nuclei and nuclear fragments beneath the central zone as well as in the bow region. As in the vital dye study, the old lenses and the 14-month old X-irradiated lenses always contained large numbers of DNA positive material in the cortex beneath the central zone and a greatly expanded bow region. In old animals or cataractous X-rayed animals the debris sometimes filled the whole outer cortex of the lens. For this analysis, the area beneath the “central zone” of the lens was set arbitrarily at the width of the lens nucleus. Figure 9A depicts the total number of nuclear fragments in the entire outer cortex. The total number of DNA fragments increased steadily and significantly with age in unirradiated lenses (Figure 9A), but the 14 month X-irradiated lenses (11 months post-irradiation) did not have more total cortical nuclei than age-matched unirradiated controls although the abnormal cortical nuclei were spread over a larger part of the cortex. This is probably due to the presence of fewer layers of secondary fiber cells in X-irradiated lenses as a result of X-ray-induced damage to LEC replication. In support of this possibility we measured the depth that the nuclei in the bow regions extended below the surface (from the anterior surface to the deepest fragments). As expected, the bows in the 14-month old X-rayed mice did not penetrate as deeply into the cortex as those of the age-matched controls, again probably as a result of X-ray damage to the LECs [46]. The nuclei in the bows of 14-month old X-rayed mice lenses extended only 284±19 microns into the cortex compared to 369±21 microns for age-matched controls (p<0.005) compared to only 138 microns in 3-month-old unirradiated controls and 522±15 microns in the 24-month-old mice lenses.

Importantly, however, when the number of nuclear DNA fragments in the abnormal position beneath the central zone, were compared, the X-irradiated lenses were seen to have far more than age-matched unirradiated controls. This is depicted in Figure 9B. Both 12-month (p<0.01) and 14-month X-rayed lenses (p<0.005) had significantly more DNA fragments in the cortex lying beneath the central zone than age-matched unirradiated controls. This is far from the sub-equatorial region where such nuclear fragments normally first appear.

**Figure 10:**
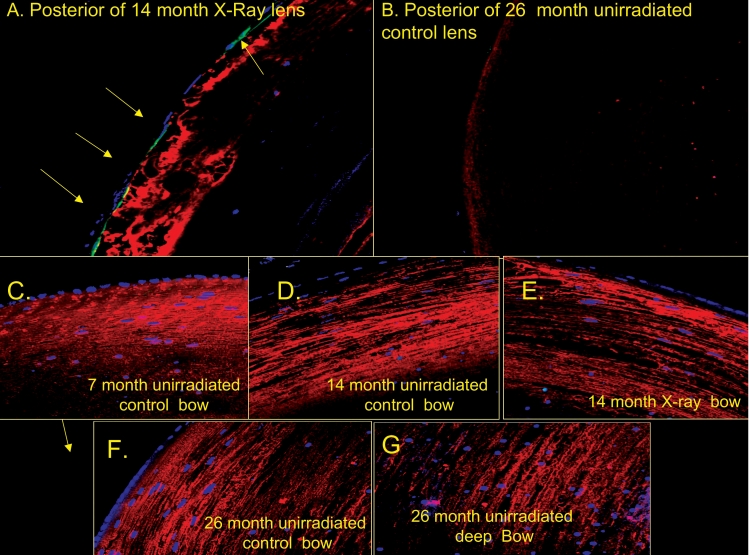
Fixed-eye mouse lens sections were stained with; antibodies to alpha smooth muscle actin shown in green, and aquaporin 0 shown in red and also DNA shown in blue as described in the Methods. Typical staining of lens fibers with aquaporin O antibodies (red) and myofibroblast-like cells (green) are shown. Note that aquaporin O comprises approximately 60% of lens fiber membrane proteins, and that myofibroblasts are normally not present in the normal young mouse lens (see text). **A**: Posterior of 14-month X-irradiated lens, arrows point out myofibroblasts (green). **B**: Posterior region of typical old (26-month) mouse lens lacking myofibroblasts. Panels **C**-**G** show images of the bow regions of irradiated or unirradiated control lenses of various ages that were all negative for myofibroblasts. Original magnification was 200×.

One unique difference between the 14-month-old X-irradiated mice lenses and the unirradiated controls was that posterior surface of all of the frequently invaded by myofibroblast-like cells that stained positive with an antibody to alpha smooth muscle actin, a marker for such cells (Figure 10A-G) [47-49]. These serve as a positive control for the absence of such from the anterior of irradiated young or old control lenses. Myofibroblast-like cells were not seen in the any of the unirradiated lenses examined, even old cataractous lenses (compare Figure 10A to Figure 10B-G). Aquaporin 0 was used as a counter stain to better visualize the lens fibers, see Figure 10A-G, since it constitutes ~60% of lens fiber membrane proteins [50,51]. Figure 10C-G also shows that the very flattened LEC nuclei in the upper bow are lodged in single secondary fibers. The more abnormal rounded nuclei lower in the bow do not appear to be as clearly sandwiched within single lens fibers, but clearly appear to be derived from the flattened nuclei closer to the surface. These spherical nuclear fragments are greatly increased in the bow and are also seen beneath the anterior central zone of both unirradiated old and X-irradiated lenses (see Figure 8).

**Figure 11:**
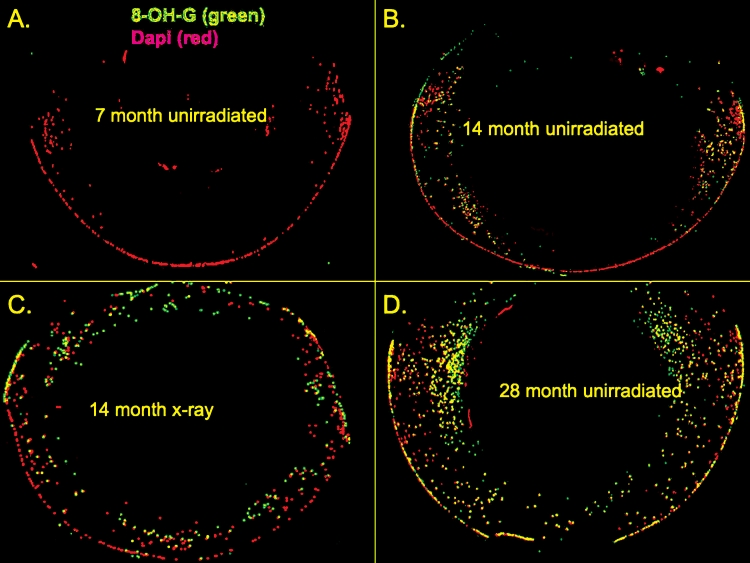
Typical panoramas of fixed-eye lens sections stained with antibodies to 8-OH-G lesions and also stained for total DNA. Fixed-eye lens sections were prepared and stained with antibodies to 8-OH guanosine and 8-OH deoxyguanosine (green), and Dapi (red) as described in the Methods. The sections were treated with 2N HCl to remove 8-OH G in RNA leaving only 8-OH deoxyguanosine residues. Head-only X-irradiation was at 3 months. The non-lens parts of the eye have been blacked out for clarity. Original magnification was 200×.

**Figure 12:**
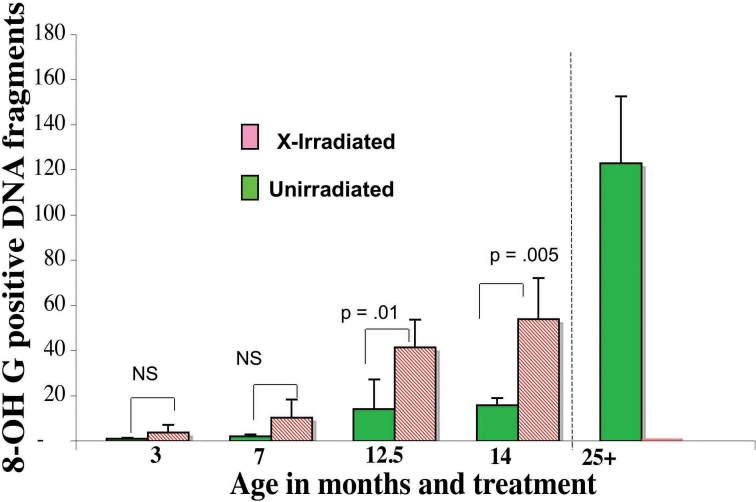
The number of 8-OH-dG positive cortical nuclear fragments found beneath the central zone. Fixed lens sections were stained with antibodies to 8-OH-dG (green) and Dapi (Red) as described in the methods (see also figure 11). The number of 8-OH-dG positive nuclear fragments resistant to 2N HCl lying only in the cortex below the Central Zone were counted and compared. The 8-OH-dG positive fragments (green) increased with age and were significantly higher in the X-irradiated lenses at 12.5 months and 14 months. Five to 7 of samples were analyzed at each age, except the 3-month X-ray with 2 mice lenses; and the 7-month X-ray with 4.

It was not possible to measure oxidized DHR or other measures of ROS activity in fixed lenses, but ROS damage was measured using an antibody to 8-hydroxy guanosine (8-OH-dG), an oxidative adduct considered to be a marker for DNA oxidative damage [52-54]). Such 8-OH-dG lesions were seen in the DNA fragments in the lens cortex of old unirradiated mouse lenses and in X-irradiated lenses but not in any of the younger control groups (Figure 11 and Figure 12).

## Discussion

Using DNA and ROS specific vital fluorescent dyes, and laser scanning confocal microscopy we have previously described 4 changes in aging rodent lenses: 1) a significantly decreased density of surface LECs in old compared to younger lenses in both mice and rats; 2) strong increases in cortical nuclei and DNA fragments in the secondary lens fibers of old rodent lenses; 3) increased cortical ROS in old rodent lenses; and 4) increased cataract concomitantly with the cortical DNA and ROS increases [12,19,20]. In the current study we have directly compared staining of age-related cataracts in mice to X-ray induced cataracts in the same mice, for the same above 4 changes. The staining for LEC density and internal DNA fragments were done using two different methods: 1) vital stains on fresh metabolizing lenses, as previously described [19,20], or 2) by staining fixed-eye lens sections for LEC density and cortical DNA with DAPI, and for ROS damage sites using an antibody to 8-OH-dG. The latter is a biomarker for oxidative damage to DNA [55]. Using both methods we find a remarkable similarity between the alterations seen in X-irradiated lenses and in the lenses from unirradiated old-mouse controls.

The LECs are the major site of cell metabolism, detoxification of ROS, transport of water, glucose, and ions into and out of the lens [2], and In addition most of the mitochondrial O_2_ consumption and ATP production occurs in the LECs and first layer or two of fiber cells [16,56-59]. The decrease in LEC density of over 40% that we found in both young X-irradiated and old unirradiated control lenses could be expected to alter the lens homeostasis in several ways including: the flow of water and ions into and out of the lens, mitochondrial ATP production and O_2_ usage; and detoxification by glutathione formation. These could all predispose to cataract formation [2,60-62]. This study supports the idea that severe decreases in LEC density precedes cataract formation in old mouse lenses, as well as, in X-irradiated mice, although the exact extent of LEC loss required to produce cataracts in rodents or humans has not been determined [2]. Earlier workers have also reported damage to and/or loss of LECs following X-radiation and other forms of oxidative insult (see review by Spector [6]) to lenses of mice, rats, rabbits, and frogs [6,22-28,30-32,63-65]. Worgul and others have proposed that at low to moderate X-irradiation doses, cataract formation is dependent on differentiation of surviving LECs into defective fibers (see review by Worgul [29). Our current work is consistent with that hypothesis, but also presents evidence that age-related cortical cataract also follows a similar course of primary damage to the surface LECs, followed by differentiation into defective secondary fiber cells with aberrant migration into the cortex and unable to degrade nuclei and possibly other organelles.

The loss of nuclei and organelles is believed to be necessary to ensure the transparency of the lens [10,11,13,15,66]. We note that a decreased ability to degrade lens fiber organelles has been shown to result in the development of cataract in several different rodent models [1,67,68]. Importantly, the degradation of DNA in differentiating lens fibers requires the lysosomal enzyme, DNase II beta (DLAD), and in DLAD knock out mice nuclei are not degraded, resulting in lens opacity and cataract formation [10,11,69-71]. Together these studies indicate that failure of the lens to digest organelles and/or macromolecules such as those reported here would be likely to contribute to cataract development. Furthermore, an increase in undegraded DNA may reflect a generalized loss of lysosomal and/or proteosomal activity in the old and X-irradiated lens, which may also predispose the lenses to the formation of opacities [1,8]. These events may precede or be causal for the aggregation and oxidation of lens proteins that are found in cataract opacities [3,72,73].

Previously, we ascribed the increased cortical DNA beneath the central zone to either an expansion of the bow or “involutions” of LECs beneath the anterior surface [12]. The fixed-eye studies in this manuscript indicate that the majority of undigested cortical nuclei arise from an expansion of the bow region rather than direct invasion of LECs from the surface in both normal aging and in X-ray induced cataracts. This is in agreement with earlier reports on X-ray induced cataracts (see review by Worgul [29]). However, frequent gaps can be seen in fixed eye lens sections from both the old control and X-irradiated lenses; and beneath some of these gaps, epithelial fragments are seen deep beneath the surface (unpublished observations). Therefore, LECs detaching from the outer epithelial layer may contribute to the abnormal DNA fragments seen in the mid anterior cortex. The bow region in unirradiated old control and X-rayed lenses can be seen to extend far into the posterior cortex in fixed-eye lens sections; and by 11 months post X-ray, some mouse lenses contained cortical DNA completely encircling the lens nucleus (compare Figure 8A-D).

There were some distinct differences between X-ray induced and age-related lens changes. Notably, the posterior surface of 14 month X-irradiated lenses were heavily populated by myofibroblast-like cells that were stained with antibodies to alpha-smooth muscle actin (see Figure 10). Alpha smooth muscle actin positivity of cells in the epithelia has been reported to occur in some cataract models associated with TGF-beta overexpression, or alkalai burn damage to the epithelial surface [47-49,74,75]. Such myofibroblast-like cells were only seen on the posterior of X-irradiated mouse lenses. We did not observe any alpha-smooth muscle positive cells on the anterior surface of X-irradiated lenses, or anywhere on the oldest unirradiated mouse lenses. So transdifferentiation of LECs to myofibroblast-like cells does not appear to be causal to the accumulation of cortical nuclei and nuclear fragments in either ARC, or X-ray induced cataractous lenses.

Excessive ROS has been proposed as one probable initiator of damage to the lens macromolecules that leads to precipitation of lens proteins and cataracts [1]. High ROS not only directly damages macromolecules in the lens, but also depletes glutathione in the lens increasing oxidative injuries further [38]. We previously reported high levels of ROS in old cataractous mouse and rat lenses stained vitally with DHR [19,20]. In this study we observed similar heightened ROS in cataractous X-irradiated mice beginning about 6–7 months after X-irradiation (Figure 5 and Figure 6). X-irradiation would be expected to directly produce short-lived ROS in addition to damage by direct energy transfer to the lens macromolecules. However, significant increases in ROS were not seen in irradiated lenses for the first 3 months after X-irradiation, but rather, did not appear until 6 months following X-irradiation, and further, must have been produced by some continuous process such as damaged mitochondria or other sources [1,6,76-78].

Such excess ROS would be expected to damage the DNA, producing 8-OH-dG adducts [55]. We used an antibody to 8-OH-dG lesions in DNA [53,55,79] to reveal these damage sites in fixed-eye sections. We now report that both control unirradiated-old and late X-irradiated cataractous mouse lenses (9–11 months post X-irradiation at 3 months of age) were positive for 8-OH-dG adducts in the accumulated cortical DNA fragments (see Figure 11 and Figure 12). We suggest that the imaged 8-OH-dG lesions are indeed in DNA, not 8-OH-G in RNA, because the antibody localizes in the nuclei and dapi stained fragments of DNA not in the cytoplasm, and the method for denaturing the DNA before staining (treatment with 2 N HCl) destroys RNA. The 8-OH-dG positive DNA fragments were more frequent in the abnormally positioned (anterior and posterior and deepest) fragments (see Figure 11). There was also a trend toward increased 8-OH-dG adducts in the surface epithelia of unirradiated old lenses (24 months+) compared to 7 month or 3 months old unirradiated lenses (p<0.05 by *t*-test. not shown), but this was not significant by the non-parametric Mann–Whitney test.

We have previously proposed [12,19,20] that the primary age-related loss of LECs may result in decreased mitochondrial respiration in or near the lens surface. As a result O_2_ levels may rise inside the cortex, resulting in the ROS noted above. Penetration of O_2_ deeply into the lens cortex would likely compromise internal stores of glutathione and other internal lens antioxidants, raising damage done by ROS and leading to protein aggregation and cataract [43,58,59,80]. Of course, as discussed above, the loss of surface epithelia may damage the lenses in several other ways including altered water, nutrient and ion flows into and out of the lens [6], and altered differentiation of damaged LECs [29].

Table 1 summarizes the findings observed in irradiated mouse lenses compared to those that occur in old unirradiated mouse lenses. The changes we observed following irradiation with X-rays occurred more rapidly but in the same 4 stages as previously shown to occur during aging of rodent lenses. These changes in rodent lenses may contribute to cataract development in both ARC and X-irradiated young mice and by the same process.

**Table 1:** Summary of similarities and differences between unirradiated old-mouse lenses and 14-month old mouse lenses X-irradiated at 3 months.

**Type of change**	Changes in X-irradiated* mice	**Reference**
Surface LEC density	Decreased by 30%–40%	This study [29],
Cataract incidence	Increased to grade 3.5	This study [12,29],
Cortical nuclei, debris, DNA	Up over 300%	This study [29]
ROS in cortex	Up 4,000%	This study
8-OH-dG staining for ROS damage to DNA	Cortical nuclear fragments positive for 8-OH-dG	This study
Alpha smooth muscle actin staining cells	Present only in myofibroblasts on posterior surface by 7–11 months post X-ray.	This study
**Type of change**	**Changes seen in unirradiated old mice (at 24–29 months)**	**Reference**
Surface LEC density	Down 30%–40% by 24–32 months.	This study [19,20],
Cataract incidence	Up ~700% by 24–32 months	[19,20]
Internalized nuclei, debris, DNA	Up 350% by 24–32 months	This study [19,20],
ROS in cortex	Up −400% by 24–32 months	This study [20] (measured in rat)
8-OH-dG positive cortical nuclei	Cortical nuclear fragments positive by 24–32 months	This study
Alpha smooth muscle actin staining for myofibroblasts	None seen	This study

A grade 3.5–4 indicates mature cataract. The asterisk indicates mice given 11 Gy of head-only radiation. In slit lamp examinations the transition from grade 1 to grade 3.5 occurs.

## References

[1] Vrensen GF Early cortical lens opacities: a short overview. Acta Ophthalmol 2009 87 602 10 19719805 10.1111/j.1755-3768.2009.01674.x

[2] Bhat SP The ocular lens epithelium. Biosci Rep 2001 21 537 63 11900326 10.1023/a:1017952128502

[3] Clark JI Order and disorder in the transparent media of the eye. Exp Eye Res 2004 78 427 32 15106922 10.1016/j.exer.2003.10.008

[4] Berthoud VM Beyer EC Oxidative stress, lens gap junctions, and cataracts. Antioxid Redox Signal 2009 11 339 53 18831679 10.1089/ars.2008.2119 PMC2763361

[5] Spector A Oxidation and aspects of ocular pathology. CLAO J 1990 16 S8 10 2407387

[6] Spector A Oxidative stress-induced cataract: mechanism of action. FASEB J 1995 9 1173 82 7672510

[7] Varma SD Devamanoharan PS Morris SM Prevention of cataracts by nutritional and metabolic antioxidants. Crit Rev Food Sci Nutr 1995 35 111 29 7748471 10.1080/10408399509527691

[8] Vrensen GF Aging of the human eye lens–a morphological point of view. Comp Biochem Physiol A Physiol 1995 111 519 32 7671147 10.1016/0300-9629(95)00053-a

[9] Bhuyan DK Bhuyan KC Assessment of oxidative stress to eye in animal model for cataract. Methods Enzymol 1994 233 630 9 8015495 10.1016/s0076-6879(94)33066-2

[10] Bassnett S On the mechanism of organelle degradation in the vertebrate lens. Exp Eye Res 2009 88 133 9 18840431 10.1016/j.exer.2008.08.017 PMC2693198

[11] De Maria A Bassnett S DNase IIbeta distribution and activity in the mouse lens. Invest Ophthalmol Vis Sci 2007 48 5638 46 18055814 10.1167/iovs.07-0782

[12] Wolf N Pendergrass W Singh N Swisshelm K Schwartz J Radiation cataracts: mechanisms involved in their long delayed occurrence but then rapid progression. Mol Vis 2008 14 274 85 18334943 PMC2254966

[13] Bassnett S Lens organelle degradation. Exp Eye Res 2002 74 1 6 11878813 10.1006/exer.2001.1111

[14] Bassnett S Kuszak JR Reinisch L Brown HG Beebe DC Intercellular communication between epithelial and fiber cells of the eye lens esterases. J Cell Sci 1994 107 799 811 8056837 10.1242/jcs.107.4.799

[15] Bassnett S Fiber cell denucleation in the primate lens. Invest Ophthalmol Vis Sci 1997 38 1678 87 9286256

[16] Bassnett S McNulty R The effect of elevated intraocular oxygen on organelle degradation in the embryonic chicken lens. J Exp Biol 2003 206 4353 61 14581604 10.1242/jeb.00670

[17] Bassøe CF Li N Ragheb K Lawler G Sturgis J Robinson JP Investigations of phagosomes, mitochondria, and acidic granules in human neutrophils using fluorescent probes. Cytometry 2003 51 21 9 12500294 10.1002/cyto.b.10003

[18] Michael R van Marle J Vrensen GF van den Berg T Changes in the refractive index of lens fibre membranes during maturation–impact on lens transparency. Exp Eye Res 2003 77 93 9 12823992 10.1016/s0014-4835(03)00065-4

[19] Pendergrass W Penn P Possin D Wolf N Accumulation of DNA, nuclear and mitochondrial debris, and ROS at sites of age-related cortical cataract in mice. Invest Ophthalmol Vis Sci 2005 46 4661 70 16303963 10.1167/iovs.05-0808

[20] Pendergrass WR Penn PE Possin DE Wolf NS Cellular debris and ROS in age-related cortical cataract are caused by inappropriate involution of the surface epithelial cells into the lens cortex. Mol Vis 2006 12 712 24 16807531

[21] Wolf N Penn P Pendergrass W Van Remmen H Bartke A Rabinovitch P Martin GM Age-related cataract progression in five mouse models for anti-oxidant protection or hormonal influence. Exp Eye Res 2005 81 276 85 16129095 10.1016/j.exer.2005.01.024

[22] Cogan DG Donaldson DD Goff JL Graves E Experimental radiation cataract. III. Further experimental studies on X-ray and neutron irradiation of the lens. AMA Arch Opthalmol 1953 50 597 602 13091536

[23] Hanna C O'Brien JE Lens epithelial cell proliferation and migration in radiation cataracts. Radiat Res 1963 19 1 11 13952544

[24] Merriam GR Jr Szechter A The effect of age on the radiosensitivity of rat lenses. Trans Am Ophthalmol Soc 1973 71 88 108 10949592 PMC1310484

[25] Worgul BV Rothstein H Congenital cataracts associated with disorganized meridional rows in a new laboratory animal: the degu (Octodon degus). Biomedicine 1975 23 1 4 1174633

[26] Worgul BV Merriam GR Szechter A Srinivasan D Lens epithelium and radiation cataract. I. Preliminary studies. Arch Ophthalmol 1976 94 996 9 938288 10.1001/archopht.1976.03910030506013

[27] Rini FJ Worgul BV Merriam GR Jr Radiation cataracogenesis in rat lenses. Bull N Y Acad Med 1986 62 744 53 3464324 PMC1629123

[28] Holsclaw DS Merriam GR Jr Medvedovsky C Rothstein H Worgul BV Stationary radiation cataracts: an animal model. Exp Eye Res 1989 48 385 98 2784389 10.1016/s0014-4835(89)80007-7

[29] Worgul BV Merriam GR Jr Medvedovsky C Cortical cataract development–an expression of primary damage to the lens epithelium. Lens Eye Toxic Res 1989 6 559 71 2487271

[30] Richards RD Changes in lens epithelium after x-ray or neutron irradiation (mouse and rabbit). Trans Am Ophthalmol Soc 1966 64 700 34 5964939 PMC1310251

[31] Richards RD Michaelis M X-ray injury to lens epithelium: effect of half-shielding on acid phosphatase in rabbits. Exp Eye Res 1971 12 293 7 5130272 10.1016/0014-4835(71)90152-7

[32] Richards RD Schocket SS Michaelis M Histochemical changes in lens epithelium of rabbits after x–irradiation. Invest Ophthalmol 1970 9 116 21 5415016

[33] Hawkins RB Mammalian cell killing by ultrasoft X rays and high-energy radiation: an extension of the MK model. Radiat Res 2006 166 431 42 16881744 10.1667/RR3594.1

[34] Gollapalle E Wang R Adetolu R Tsao D Francisco D Sigounas G Georgakilas AG Detection of oxidative clustered DNA lesions in X-irradiated mouse skin tissues and human MCF-7 breast cancer cells. Radiat Res 2007 167 207 16 17390728 10.1667/rr0659.1

[35] Fan R Kumaravel TS Jalali F Marrano P Squire JA Bristow RG Defective DNA strand break repair after DNA damage in prostate cancer cells: implications for genetic instability and prostate cancer progression. Cancer Res 2004 64 8526 33 15574758 10.1158/0008-5472.CAN-04-1601

[36] Risom L Moller P Vogel U Kristjansen PE Loft S X-ray-induced oxidative stress: DNA damage and gene expression of HO-1, ERCC1 and OGG1 in mouse lung. Free Radic Res 2003 37 957 66 14670003 10.1080/1071576031000150788

[37] Miura Y Anzai K Urano S Ozawa T In vivo electron paramagnetic resonance studies on oxidative stress caused by X-irradiation in whole mice. Free Radic Biol Med 1997 23 533 40 9215798 10.1016/s0891-5849(97)00103-2

[38] Giblin FJ Chakrapani B Reddy VN The effects of X-irradiation on lens reducing systems. Invest Ophthalmol Vis Sci 1979 18 468 75 35484

[39] Melov S Wolf N Strozyk D Doctrow SR Bush AI Mice transgenic for Alzheimer disease beta-amyloid develop lens cataracts that are rescued by antioxidant treatment. Free Radic Biol Med 2005 38 258 61 15607908 10.1016/j.freeradbiomed.2004.10.023

[40] Maurya OP Mohanty L Bhaduri G Chandra A Role of anti-oxidant enzymes superoxide dismutase and catalase in the development of cataract: study of serum levels in patients with senile and diabetic cataracts. J Indian Med Assoc 2006 104 396 7 17240813

[41] Vinson JA Oxidative stress in cataracts. Pathophysiology 2006 13 151 62 16765571 10.1016/j.pathophys.2006.05.006

[42] Marsili S Salganik RI Albright CD Freel CD Johnsen S Peiffer RL Costello MJ Cataract formation in a strain of rats selected for high oxidative stress. Exp Eye Res 2004 79 595 612 15500819 10.1016/j.exer.2004.06.008

[43] Truscott RJ Age-related nuclear cataract-oxidation is the key. Exp Eye Res 2005 80 709 25 15862178 10.1016/j.exer.2004.12.007

[44] Pendergrass W Wolf N Poot M Efficacy of MitoTracker Green and CMXrosamine to measure changes in mitochondrial membrane potentials in living cells and tissues. Cytometry 2004 61 162 9 15382028 10.1002/cyto.a.20033

[45] Ohno M Oka S Nakabeppu Y Quantitative analysis of oxidized guanine, 8-oxoguanine, in mitochondrial DNA by immunofluorescence method. Methods Mol Biol 2009 554 199 212 19513676 10.1007/978-1-59745-521-3_13

[46] Riley EF Miller RC Lindgren AL Recovery of murine lens epithelial cells from single and fractionated doses of X rays and neutrons. Radiat Res 1988 114 567 78 3375443

[47] Lovicu FJ Steven P Saika S McAvoy JW Aberrant lens fiber differentiation in anterior subcapsular cataract formation: a process dependent on reduced levels of Pax6. Invest Ophthalmol Vis Sci 2004 45 1946 53 15161862 10.1167/iovs.03-1206

[48] Lovicu FJ Ang S Chorazyczewska M McAvoy JW Deregulation of lens epithelial cell proliferation and differentiation during the development of TGFbeta-induced anterior subcapsular cataract. Dev Neurosci 2004 26 446 55 15855773 10.1159/000082286

[49] Shirai K Saika S Tanaka T Okada Y Flanders KC Ooshima A Ohnishi Y A new model of anterior subcapsular cataract: involvement of TGFbeta/Smad signaling. Mol Vis 2006 12 681 91 16807527

[50] Grey AC Li L Jacobs MD Schey KL Donaldson PJ Differentiation-dependent modification and subcellular distribution of aquaporin-0 suggests multiple functional roles in the rat lens. Differentiation 2009 77 70 83 19281766 10.1016/j.diff.2008.09.003 PMC2696237

[51] Broekhuyse RM Kuhlmann ED Winkens HJ Lens membranes VII. MIP is an immunologically specific component of lens fiber membranes and is identical with 26K band protein. Exp Eye Res 1979 29 303 13 118041 10.1016/0014-4835(79)90009-5

[52] Nunomura A Tamaoki T Tanaka K Motohashi N Nakamura M Hayashi T Yamaguchi H Shimohama S Lee HG Zhu X Smith MA Perry G Intraneuronal amyloid beta accumulation and oxidative damage to nucleic acids in Alzheimer disease. Neurobiol Dis 2010 37 731 7 20034567 10.1016/j.nbd.2009.12.012 PMC2825655

[53] Moreira PI Sayre LM Zhu X Nunomura A Smith MA Perry G Detection and localization of markers of oxidative stress by in situ methods: application in the study of Alzheimer disease. Methods Mol Biol 2010 610 419 34 20013193 10.1007/978-1-60327-029-8_25 PMC5914163

[54] Sayre LM Perry G Smith MA In situ methods for detection and localization of markers of oxidative stress: application in neurodegenerative disorders. Methods Enzymol 1999 309 133 52 10507022 10.1016/s0076-6879(99)09012-6

[55] Evans M, Cooke M. Oxidative damage to nucleic acids. Illustrated. ed. New York: Springer; 2007.

[56] Eaton JW Is the lens canned? Free Radic Biol Med 1991 11 207 13 1937139 10.1016/0891-5849(91)90173-z

[57] Holekamp NM Shui YB Beebe DC Vitrectomy surgery increases oxygen exposure to the lens: a possible mechanism for nuclear cataract formation. Am J Ophthalmol 2005 139 302 10 15733992 10.1016/j.ajo.2004.09.046

[58] Shui YB Fu JJ Garcia C Dattilo LK Rajagopal R McMillan S Mak G Holekamp NM Lewis A Beebe DC Oxygen distribution in the rabbit eye and oxygen consumption by the lens. Invest Ophthalmol Vis Sci 2006 47 1571 80 16565394 10.1167/iovs.05-1475

[59] Barbazetto IA Liang J Chang S Zheng L Spector A Dillon JP Oxygen tension in the rabbit lens and vitreous before and after vitrectomy. Exp Eye Res 2004 78 917 24 15051473 10.1016/j.exer.2004.01.003

[60] Tkachov SI Lautenschlager C Ehrich D Struck HG Changes in the lens epithelium with respect to cataractogenesis: light microscopic and Scheimpflug densitometric analysis of the cataractous and the clear lens of diabetics and non-diabetics. Graefes Arch Clin Exp Ophthalmol 2006 244 596 602 16175371 10.1007/s00417-005-0091-7

[61] Kumamoto Y Takamura Y Kubo E Tsuzuki S Akagi Y Epithelial cell density in cataractous lenses of patients with diabetes: association with erythrocyte aldose reductase. Exp Eye Res 2007 85 393 9 17655844 10.1016/j.exer.2007.06.007

[62] Hasanova N Kubo E Kumamoto Y Takamura Y Akagi Y Age-related cataracts and Prdx6: correlation between severity of lens opacity, age and the level of Prdx 6 expression. Br J Ophthalmol 2009 93 1081 4 19429582 10.1136/bjo.2008.152272

[63] Von Sallmann L The lens epithelium in the pathogenesis of cataract; the XIII Edward Jackson Memorial lecture. Am J Ophthalmol 1957 44 159 70 13444404 10.1016/0002-9394(57)90001-6

[64] Von Sallmann L Experimental studies on early lens changes after roentgen irradiation. III. Effect of x-radiation on mitotic activity and nuclear fragmentation of lens epithelium in normal and cysteine-treated rabbits. AMA Arch Opthalmol 1952 47 305 20 14902204

[65] Worgul BV Merriam GR Jr Medvedovsky C Brenner DJ Accelerated heavy particles and the lens. III. Cataract enhancement by dose fractionation. Radiat Res 1989 118 93 100 2704794

[66] Wistow GJ Piatigorsky J Lens crystallins: the evolution and expression of proteins for a highly specialized tissue. Annu Rev Biochem 1988 57 479 504 3052280 10.1146/annurev.bi.57.070188.002403

[67] Fromm L Overbeek PA Inhibition of cell death by lens-specific overexpression of bcl-2 in transgenic mice. Dev Genet 1997 20 276 87 9216067 10.1002/(SICI)1520-6408(1997)20:3<276::AID-DVG10>3.0.CO;2-6

[68] Perez-Castro AV Tran VT Nguyen-Huu MC Defective lens fiber differentiation and pancreatic tumorigenesis caused by ectopic expression of the cellular retinoic acid-binding protein I. Development 1993 119 363 75 8287793 10.1242/dev.119.2.363

[69] Nishimoto S Kawane K Watanabe-Fukunaga R Fukuyama H Ohsawa Y Uchiyama Y Hashida N Ohguro N Tano Y Morimoto T Fukuda Y Nagata S Nuclear cataract caused by a lack of DNA degradation in the mouse eye lens. Nature 2003 424 1071 4 12944971 10.1038/nature01895

[70] Nagata S DNA degradation in development and programmed cell death. Annu Rev Immunol 2005 23 853 75 15771588 10.1146/annurev.immunol.23.021704.115811

[71] Nakahara M Nagasaka A Koike M Uchida K Kawane K Uchiyama Y Nagata S Degradation of nuclear DNA by DNase II-like acid DNase in cortical fiber cells of mouse eye lens. FEBS J 2007 274 3055 64 17509075 10.1111/j.1742-4658.2007.05836.x

[72] Sharma KK Santhoshkumar P Lens aging: effects of crystallins. Biochim Biophys Acta 2009 1790 1095 108 19463898 10.1016/j.bbagen.2009.05.008 PMC2743770

[73] Sandilands A Hutcheson AM Long HA Prescott AR Vrensen G Loster J Klopp N Lutz RB Graw J Masaki S Dobson CM MacPhee CE Quinlan RA Altered aggregation properties of mutant gamma-crystallins cause inherited cataract. EMBO J 2002 21 6005 14 12426373 10.1093/emboj/cdf609 PMC137201

[74] Ruiz-Ederra J Verkman AS Accelerated cataract formation and reduced lens epithelial water permeability in aquaporin-1-deficient mice. Invest Ophthalmol Vis Sci 2006 47 3960 7 16936111 10.1167/iovs.06-0229

[75] de Iongh RU Wederell E Lovicu FJ McAvoy JW Transforming growth factor-beta-induced epithelial-mesenchymal transition in the lens: a model for cataract formation. Cells Tissues Organs 2005 179 43 55 15942192 10.1159/000084508

[76] Davies KJ Protein oxidation and proteolytic degradation. General aspects and relationship to cataract formation. Adv Exp Med Biol 1990 264 503 11 2244532 10.1007/978-1-4684-5730-8_77

[77] Taylor A Associations between nutrition and cataract. Nutr Rev 1989 47 225 34 2685667 10.1111/j.1753-4887.1989.tb02848.x

[78] Varma SD Chand D Sharma YR Kuck JF Jr Richards RD Oxidative stress on lens and cataract formation: role of light and oxygen. Curr Eye Res 1984 3 35 57 6360540 10.3109/02713688408997186

[79] Poulsen HE Loft S Early biochemical markers of effects: enzyme induction, oncogene activation and markers of oxidative damage. Toxicology 1995 101 55 64 7631323 10.1016/0300-483x(95)03016-9

[80] Giblin FJ Glutathione: a vital lens antioxidant. J Ocul Pharmacol Ther 2000 16 121 35 10803423 10.1089/jop.2000.16.121

